# Design of Nanostructured Lipid Carriers Containing *Cymbopogon martinii* (Palmarosa) Essential Oil against *Aspergillus nomius*

**DOI:** 10.3390/molecules26164825

**Published:** 2021-08-10

**Authors:** Denise Tiemi Uchida, Gabriella Ferreira Siqueira, Edson Marques dos Reis, Fábio Luis Hegeto, Antonio Medina Neto, Adriano Valim Reis, Marcos Luciano Bruschi, Mônica Villa Nova, Miguel Machinski Júnior

**Affiliations:** 1 Department of Health Basic Sciences, State University of Maringá, Maringá 87020-900, PR, Brazil; denisetiemi13@gmail.com (D.T.U.); mmjunior@uem.br (M.M.J.); 2 Department of Pharmacy, State University of Maringá, Maringá 87020-900, PR, Brazil; gabisiqueiraa9@gmail.com (G.F.S.); avreis@uem.br (A.V.R.); monica.villano@gmail.com (M.V.N.); 3 Department of Chemistry, State University of Maringá, Maringá 87020-900, PR, Brazil; emreis@uem.br; 4 Department of Physics, State University of Maringá, Maringá 87020-900, PR, Brazil; hegetofl@hotmail.com (F.L.H.); medina@dfi.uem.br (A.M.N.)

**Keywords:** biofungicides, brazil nut, geraniol, nanostructured lipid system, *Bertholletia excelsa* Humb. & Bompl

## Abstract

Palmarosa essential oil (PEO) is an alternative to synthetic fungicides to control the contamination by food-deteriorating fungi, such as *Aspergillus nomius*. Nonetheless, the low long-term stability and volatility hamper its utilization. Thus, this study aimed to develop nanostructured lipid carriers (NLCs) containing PEO to improve its stability and consequently prolong the activity against *A. nomius*. A mixture design was applied to find the best preparation conditions for antifungal activity. The characterization analyses included size measurements, zeta potential (ζ-potential), entrapment efficiency (EE), and antifungal activity (by inhibition of mycelial growth (IMG) and/or in situ test (pre-contaminated Brazil nuts) tests). The nanocarriers presented particle sizes smaller than 300 nm, homogeneous size distribution, ζ-potential of −25.19 to −41.81 mV, and EE between 73.6 and 100%. The formulations F5 and F10 showed the highest IMG value (98.75%). Based on the regression model, three optimized formulations (OFs) were tested for antifungal activity (IMG and in situ test), which showed 100% of inhibition and prevented the deterioration of Brazil nuts by *A. nomius*. The preliminary stability test showed the maintenance of antifungal activity and physicochemical characteristics for 90 days. These results suggest a promising system as a biofungicide against *A. nomius.*

## 1. Introduction

Fungal diseases that affect agricultural crops require special attention, not only for damage to health and the environment, but also because they affect the economy, particularly in developing countries. In 2020, Brazil exported approximately 8 tons of Brazil nuts (about US $20 million) [1]. However, Brazil’s exportation of this product is decreasing due to some factors, such as reduced production (by replacement of native Brazil nuts areas for other planting crops), commercialization of Brazil nuts by other countries, and delay in the improvement of extraction, storage, and export techniques regarding aflatoxin contamination [2,3,4]. In addition, Brazil has a large area under a tropical climate that favors contamination by deteriorating and toxigenic fungi, which makes it difficult to maintain grains and oilseeds under suitable conditions [5,6]. *Aspergillus nomius* is a fungus producer of aflatoxins type B and G, which is capable of contaminating food at harvest, storage, and after processing [7,8]. These aflatoxins are extremely toxic and harmful to health when chronically ingested, being classified as Group 1 (carcinogenic to humans) by IARC (International Agency for Research on Cancer) [9]. To prevent and control the attack of these fungi, the agricultural sector is encouraged to make use of synthetic fungicides. Nevertheless, the exacerbated use of these compounds represents a risk for non-target organisms and for the environment, in addition to causing fungi resistance [10,11]. In order to reduce the negative impacts on the environment and human and animal health, some natural and biodegradable alternatives have been studied to replace synthetic fungicides, for example, the use of essential oils [12,13].

Palmarosa (*Cymbopogon martinii*) is a perennial and aromatic plant, whose leaves and flowers produce an essential oil (EO) considered safe by the Food and Drug Administration (FDA) [14,15]. Palmarosa essential oil (PEO) is recognized for its antifungal, antibacterial, and antioxidant properties [16,17,18]. Although very versatile, EOs contain labile and volatile compounds that evaporate or decompose easily under high temperatures, low pressure, and the presence of air and light, which shortens its useful life [19]. In this context, one of the possibilities to improve the effectiveness of EOs is the use of encapsulation techniques, such as lipid nanoparticles, which can protect these compounds from degradation and loss of biological activity [20].

Among lipid systems, there are two main types, solid lipid nanoparticles (SLNs) and nanostructured lipid carriers (NLCs) [21]. The use of NLCs has significantly increased due to their ability to encapsulate hydrophobic molecules with high efficiency, increase drug stability/availability during storage, and overcome some limitations of SLNs (e.g., comparatively lower loading capacity and core material (encapsulate) loss during storage) [22,23,24]. Considering the food industry, lipid nanoparticles have some advantages over other systems, such as biocompatibility, low toxicity, ease for large-scale production, and no need to use organic solvents to prepare the formulation [22,25]. Previous studies reported the successful loading of EOs into NLCs and showed they were efficient for protection, production of fortified food products, and as a potential natural food preservative [21,22,24,25,26,27,28]. Furthermore, since these nanoparticles are composed of lipids, they have the ability to interact with several bacterial and fungal cell types and can also facilitate the antimicrobial activity of EOs by improving the diffusion properties through biological membranes due to the nanosized and lipophilic nature [28].

Unlike SLNs, the NLC matrix is formed by a blend of solid and liquid lipids; the presence of liquid oil creates larger empty spaces through the solid lipid, which can provide higher solubility and entrap bioactive compounds [22,25]. In this work, cocoa butter and sesame oil were used as lipid materials. Considering the wide acceptance of natural products as food preservatives, such as EO, combined with the necessity of increasing their stability, the aim of this work was to develop an NLC containing PEO as a formulation to avoid the contamination of nuts by *A. nomius*. To the best of our knowledge, there is no other study involving encapsulation of PEO into NLCs with this purpose.

## 2. Results and Discussion

### 2.1. Chemical Analysis of Palmarosa Essential Oil by Gas Chromatography/Mass Spectrometry (GC-MS)

The validated GC-MS method could identify 10 constituents in the PEO, which represent a total of 99.7% of the oil composition. The major constituents (see Table 1) were geraniol (80.37%), geraniol ester (10.9%), and linalool (3.0%) (Figure S1 in Supplementary Materials shows the chromatogram of PEO by GC/MS). Some studies with PEO also reported geraniol as the major compound [17,18]. On the other hand, in other studies, the amount was lower than the one obtained in this work. For example, Devi et al. [29] found a geraniol concentration of 43.8% while Kalagatur et al. [15] found a geraniol concentration of 19.1%. These differences are normal since they are influenced by intrinsic (genetic) and extrinsic (collection site, climate, period of the year, soil composition) factors [15,30].

### 2.2. Antifungal Activity of Non-Encapsulated Palmarosa Essential Oil

The minimum inhibitory concentration (MIC) and minimum fungicidal concentration (MFC) obtained for PEO was 250 μg mL^−1^. This represents a satisfactory result, being equal to the MIC value (250 μg mL^−1^) of the synthetic fungicide Carbendazim, known for controlling infections by *Aspergillus* spp. [31]. These analyses are essential to determine the concentration of PEO that should be added to the formulations to inhibit the fungi growth. The PEO showed antifungal activity against two strains of *Aspergillus* spp., *A. tubingensis* (MIC = 2.52 μg mL^−1^) and *A. minutus* (MIC = 0.7 μg mL^−1^), in the study of Angeline et al. [32]. Kalagatur et al. [15] reported that PEO was effective against *Fusarium graminearum*, with MIC and MFC values corresponding to 421.7 ppm (421.7 μg mL^−1^) and 618.3 ppm (618.3 μg mL^−1^), respectively. Regarding *Rhizopus stolonifer*, the main fungi associated with strawberry contamination, the MIC and MFC values reported for PEO were 5 and 10 μg mL^−1^, respectively [33]. These different results indicate that the antifungal activity of PEO may vary according to the fungi species, since the antimicrobial activity promoted by EOs occurs through synergism or antagonism of their chemical constituents [34].

### 2.3. Experimental Design

After preliminary tests to establish some parameters (e.g., time and speed stirring of ultra-dispersion, concentration of Tween 80, amplitude and time for sonication, PEO:SO ratio), a mixture design (see Section 3.5) was applied to find the adequate amount of cocoa butter (3.60–4.45%), PEO:SO (0.75–1.25%, in a proportion of 1:3) and/or P80H (0.5–1%) to obtain formulations with the best results related to the antifungal activity (inhibition of mycelial growth (IMG)) against *A. nomius*. The software proposed a mixture design, which resulted in 10 formulations. In order to increase the degrees of freedom and permit the residue analysis, three formulations were replicated, resulting in a total of 13 formulations (F1–F13). For all formulations, the concentration of PEO was above the MIC/MFC values. The percentages of IMG for each NLC containing PEO are shown in Table 2. The higher the IMG values, the better the formulation can inhibit the growth of the microorganism.

**Figure 1:**
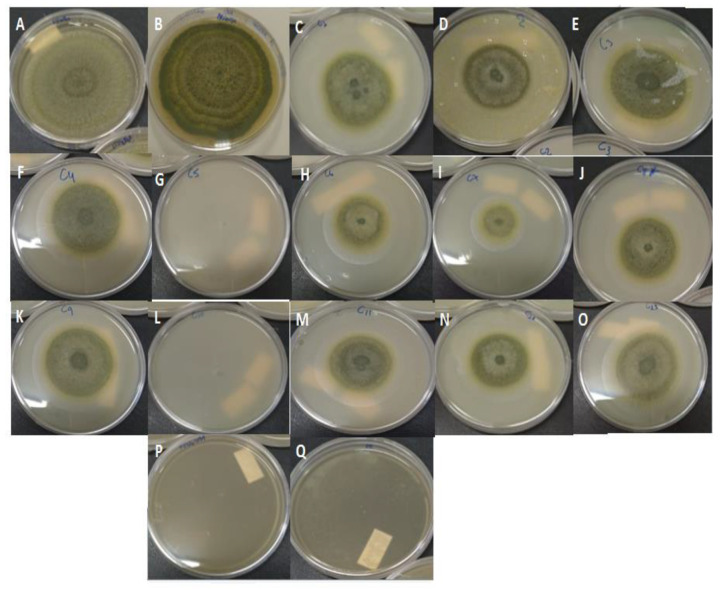
Images of Petri dishes inoculated with *A. nomius* and treated with blank NLC, PEO-loaded NLC (F1-F13), Carbendazim, or non-encapsulated PEO, after 10 days of incubation: (**A**) fungal control; (**B**) negative control (blank NLC); (**C**) F1; (**D**) F2; (**E**) F3; (**F**) F4; (**G**) F5; (**H**) F6; (**I**) F7; (**J**) F8; (**K**) F9; (**L**) F10; (**M**) F11; (**N**) F12; (**O**) F13, (**P**) positive control (Carbendazim 250 μg mL^−1^) and (**Q**) non-encapsulated PEO (250 μg mL^−1^).

Figure 1 shows the pictures of the Petri dishes after 10 days of incubation. The blank formulation (Figure 1B) did not inhibit fungi growth, indicating that only the PEO in the NLC is responsible for antifungal activity. For the formulations F5 and F10 (98.75% of IMG), mycelial growth was observed until the third day; however, it did not evolve until the 10th day, which may indicate that the PEO was released over the days and stopped the growth.

In the study of Yan et al. [35], the fungus *Rhizopus stolonifer* was incubated for a period of 24, 48, and 72 h with 3 μL of PEO in paper discs until a volatile concentration of 150 μL L^−1^ was obtained. The authors observed that the volatile compounds of the PEO could inhibit 41.6%, 24.5%, and 33.0% of the fungus growth after 24, 48, and 72 h of incubation, respectively [35]. These results demonstrated the importance of oil encapsulation because volatile compounds interfere with the effectiveness and duration of the PEO effects.

According to the statistical analysis (ANOVA), the special quartic-order Scheffe-type model (that is, a fourth order without the cubic terms) showed the best statistical significance based on the dependent variable “Y” (IMG), with an adjusted coefficient of determination (adj R^2^) of 0.9995, a signal-to-noise ratio (“Adeq Precision”) of 162.27, and no significant lack of fit, which means that the model can be used to predict IMG values (Supplementary Material, Table S1). All terms of the adopted model were significant (*p*-value < 0.05, Supplementary Materials Table S2) and, therefore, used to generate the regression model equation (Equation (1)):

(1) $$\begin{array}{cc} Y=32.29X_{1}+ & 526.29X_{2}+76.97X_{3}-757.21X_{1}X_{2}-105.06X_{1}X_{3}-998.96X_{2}X_{3}+2477.61X_{1}^{2}X_{2}X_{3}\\ & -2469.45X_{1}X_{2}^{2}X_{3}+3425.25X_{1}X_{2}X_{3}^{2} \end{array}$$

**Figure 2:**
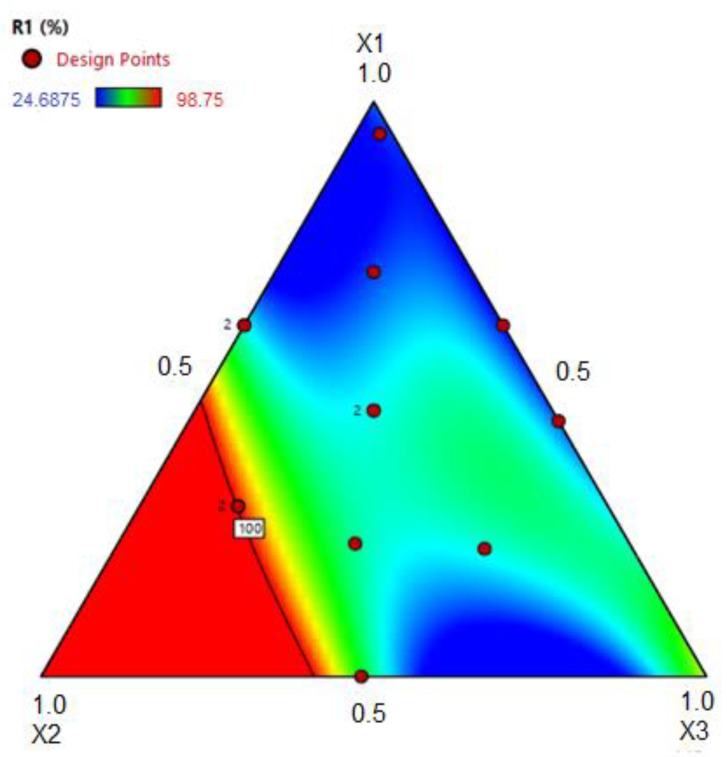
Triangular surface response for inhibition of mycelial growth (R1 %) as a function of the amount of cocoa butter (**X1**), Palmarosa essential oil:sesame oil (**X2**), and Phospholipon 80H^®^ (**X3**) given in pseudocomponents.

The effect of X1, X2, and X3 on antifungal activity (IMG = R1) is shown in Figure 2. It was observed that the highest percentage of IMG occurred when the formulation contained the highest concentration of X2 (PEO:SO), corroborated by the higher value of its coefficient in Equation (1).

### 2.4. Size, Polydispersity Index (PDI), and Zeta Potential (ζ-Potential)

The results of the size, PDI, and ζ-potential are described in Table 2. Size and PDI can influence some parameters, such as the physical and chemical stability, solubility, release rate, and biological performance [36]. The size values of NLCs ranged between 173.8 nm (F7) and 290.7 nm (F4). There is no rule that determines the size range of NLC, which can vary from 10 to 1000 nm [37]. According to some authors, NLCs with a particle size between 50 and 400 nm, the case of this study, may indicate that the components and preparation procedures were appropriate to prevent the coalescence of nanocarriers [25,38]. The PDI varied from 0.14 (F10) to 0.21 (F12). According to the literature, PDI values below 0.3 denote a homogeneous size distribution [39,40,41].

Regarding the ζ-potential, the PEO-loaded NLCs had a negative charge ranging from −25.2 (F6) to −41.8 mV (F10). This is an important and useful indicator of stability of NLCs [42,43]. Particles with ζ-potential values greater than ±30 mV are suggested to have good long-term physical stability due to the phenomenon of repulsion, decreasing the chance of particle agglomeration and the formation of precipitates [44,45].

### 2.5. Entrapment Efficiency (EE) and PEO Content (PEOC)

The EE results, obtained from a validated GC-MS-SIM method, are presented in Table 2. The lowest EE was 73.6% (F13) and the highest was 99.9% (F9). Other researchers reported similar results for NLCs containing the EO of *Cuminun cyminum*, with EE values ranging from 80 to 92% and using cocoa butter as a solid lipid as well [43]. It was demonstrated in other studies that NLCs are particularly useful for the encapsulation of lipophilic compounds, such as EO, with an EE normally greater than 70% [46,47,48,49]. The PEOC ranged from 1.37 (F13) to 3.01 (F10) mg PEO per gram of NLC.

### 2.6. Characterization of the Optimized Formulations (OFs) of Nanostructured Lipid Carriers Containing Palmarosa Essential Oil

Based on the desirability test, the regression model predicted 19 combinations of parameters X1, X2, and X3 to obtain 100% of IMG. From these 19 formulations, called optimized formulations (OFs), three (OF1, OF4, and OF12) were selected based on formulations F5 and F10, which presented the best results for IMG and EE. The composition of the optimized NLC were given in pseudocomponents and then converted into proportion and quantity (%, *w*/*w*), as shown in Table 3. The formulations were prepared according to the same procedure described in Section 3.5.

The optimized formulations were submitted to the same characterization tests (size, PI, ζ-potential, EE, and IMG). The results are presented in Table 4. These formulations exhibited a smaller particle size than the previously described NLC, maintaining PDI values below 0.3. In addition to a homogeneous size distribution, it may suggest that the compatibility between the components of the formulation was not affected and there is a lower tendency to coalescence [39,40,41]. A smaller particle size for the optimized formulation was also reported by Outuki et al. [27], who attributed the smaller size to the use of a lower concentration of P80H, corroborating the results obtained in this work. The ζ-potential was close to −30 mV, suggesting good physical stability [44].

The optimized NLC presented higher EE values (approximately 100%), which demonstrates that the materials and the preparation method were efficient at encapsulating the PEO. The PEO content was 3.17 ± 0.03; 3.15 ± 0.03, and 3.22 ± 0.07 mg g^−1^ of NLC for OF1, OF4, and OF12, respectively. Concerning the antifungal activity, OF4 and OF12 showed better results (100% of IMG), proving the predictability of the regression model, while OF1 presented a value of 97.3%. The optimized blank formulations (negative control) showed 0% of IMG, like the fungal control (FC).

**Figure 3:**
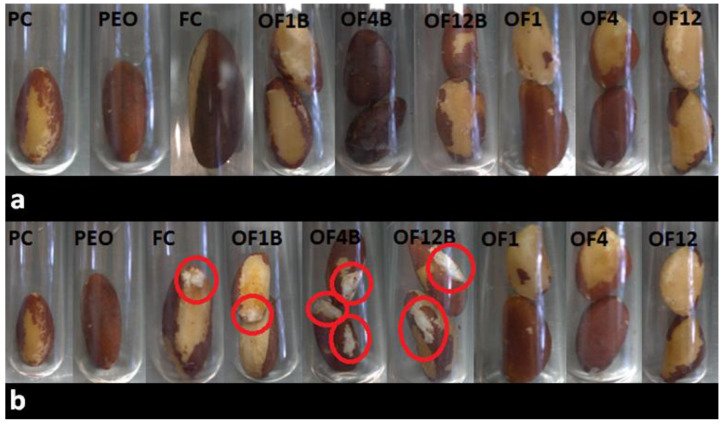
In situ antifungal activity test with Brazil nuts on the 1st day (**a**) and 10th day (**b**) after treatment. PC: positive control (Carbendazim at 250 µg mL^−1^); PEO: non-encapsulated Palmarosa essential oil (250 µg mL^−1^); FC: fungal control; OF1B: blank optimized formulation 1; OF4B: blank optimized formulation 4; OF12B: blank optimized formulation 12; OF1: optimized formulation 1; OF4: optimized formulation 4; OF12: optimized formulation 12.

The three optimized NLC formulations were tested in situ using Brazil nuts previously inoculated with *A. nomius* (see Section 3.8). In Figure 3a, which represents the first day after the treatments, the nuts did not show any signs of deterioration caused by the fungus. However, on the 10th day (Figure 3b), the FC tube (i.e., Brazil nuts treated with 0.9% saline) and those containing nuts treated with the blank formulations OF1B, OF4B, and OF12B (negative control) showed deterioration (red circle) caused by the fungus. Like the PC (Carbendazim) and the non-encapsulated PEO treatments, the optimized formulations containing PEO were able to inhibit the deterioration of Brazil nuts caused by *A. nomius*. This finding corroborates the IMG results, in which the optimized NLC could inhibit 97.3 to 100% of the fungus growth. The results for the blank NLCs (without PEO) showed that the antifungal activity depends only on the presence of PEO and not on the other formulation components.

The developed NLCs were efficient in encapsulating the PEO and the preparation process did not harm its biological activity. The effective antifungal activity of NLC containing PEO may be due to the synergism between the fungus cell membrane and the characteristic of the NLC emulsion, and/or the internalization of PEO droplets in microbial membranes by passive transport due to the size of the particle [50,51].

### 2.7. Stability Study

The size, PDI, ζ-potential, EE, pH, IMG, and in situ antifungal test with Brazil nuts of the three optimized NLC (OF1, OF4, and OF12) were evaluated for 90 days (Table 5). The physical stability was verified by comparing the macroscopic aspect of the newly prepared formulations (24 h) and after 30, 60, and 90 days of storage at room temperature (Supplementary Materials, Figure S2). There was no sign of phase separation nor formation of agglomerates during the period investigated. The pH value did not change after storage for three months (Table 5). The small particle size (between 50 and 400 nm) is one of the factors that could have contributed to the physical stability of the formulations by preventing coalescence and gravitational separation [25,38].

On the other hand, statistically significant changes in particle size, PDI, and ζ-potential were observed as a function of time for the OF1 and OF4, and in particle size for the OF12 sample. Nonetheless, the particle size and PDI for all formulations were lower than 200 nm and 0.3, respectively, and the ζ-potential results were close to −30 mV after 90 days of storage. In a study involving NLC containing cinnamon essential oil, prepared with cocoa butter as a solid lipid and different types of liquid oils (corn, sesame, almond, and black seed oil), significant changes of the particle size and PDI during storage (40 days) were also shown [26]. In another study in which cocoa butter and sesame oil were also used to prepare NLC, the PDI showed significant differences during storage (12 months at room temperature) but without losing the monodispersed size distribution (PDI < 0.18), as seen in this study.

Regarding the EE, from 24 h to 90 days, it was close to 100% for all optimized formulations. The results corroborate those found by Ribeiro et al. [52], in which the authors reported that formulations composed of cocoa butter as the solid lipid, probably due to the polymorphic forms, contributed to high EE values. No statistical difference (*p* > 0.05) was found in terms of EE (for OF4 and OF12) and pH (OF1, OF4, and OF12). Concerning the IMG test, the values were not modified within 90 days for OF4 and OF12. In general, the results for all systems were satisfactory, reinforcing the stability of the colloidal dispersions.

**Figure 4:**
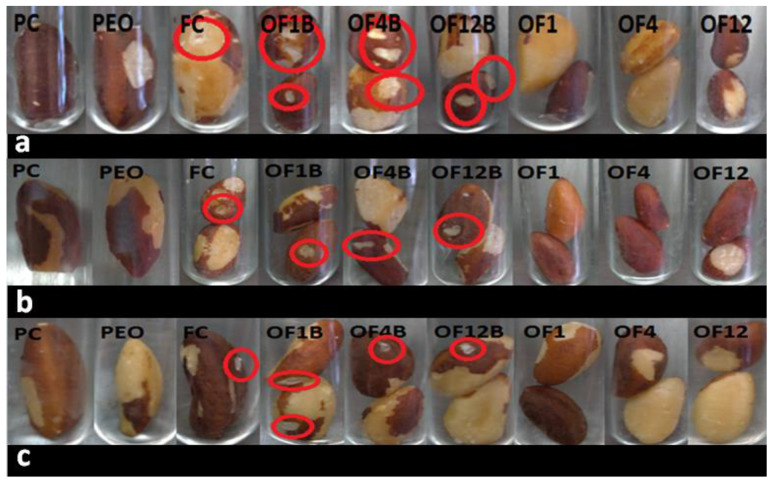
In situ antifungal activity with Brazil nuts. (**a**): 30th day of stability (10th day after treatment); (**b**): 60th day of stability (10th day after treatment); (**c**): 90th day of stability (10th day after treatment). PC: positive control (Carbendazim at 250 µg mL^−1^); PEO: non-encapsulated Palmarosa essential oil (250 µg mL^−1^); FC: fungal control; OF1B: blank optimized formulation 1; OF4B: blank optimized formulation 4; OF12B: blank optimized formulation 12; OF1: optimized formulation 1; OF4: optimized formulation 4; OF12: optimized formulation 12.

The antifungal activity test using Brazil nuts showed that the optimized formulations containing PEO were still able to inhibit the deteriorating action of *A. nomius* after 90 days of storage (Figure 4). Therefore, the physical, chemical, and microbiological stability of the formulations was demonstrated, confirming that the melt-emulsification method is suitable for preparing PEO-loaded NLC and these systems contribute to the protection of the active compound against oxidation and maintenance of antifungal activity even after 90 days.

Although the three optimized formulations were stable, only the OF12 was selected for morphological analysis by transmission electron microscopy since it showed more consistent results related to the size measurements and ζ-potential (no statistical difference) over time. In addition, OF12 was submitted to Fourier transform Raman spectroscopy analysis (the results for OF1 and OF4 are shown in Figure S3 in the Supplementary Materials).

### 2.8. Fourier Transform Raman Spectroscopy (FT-Raman)

**Figure 5:**
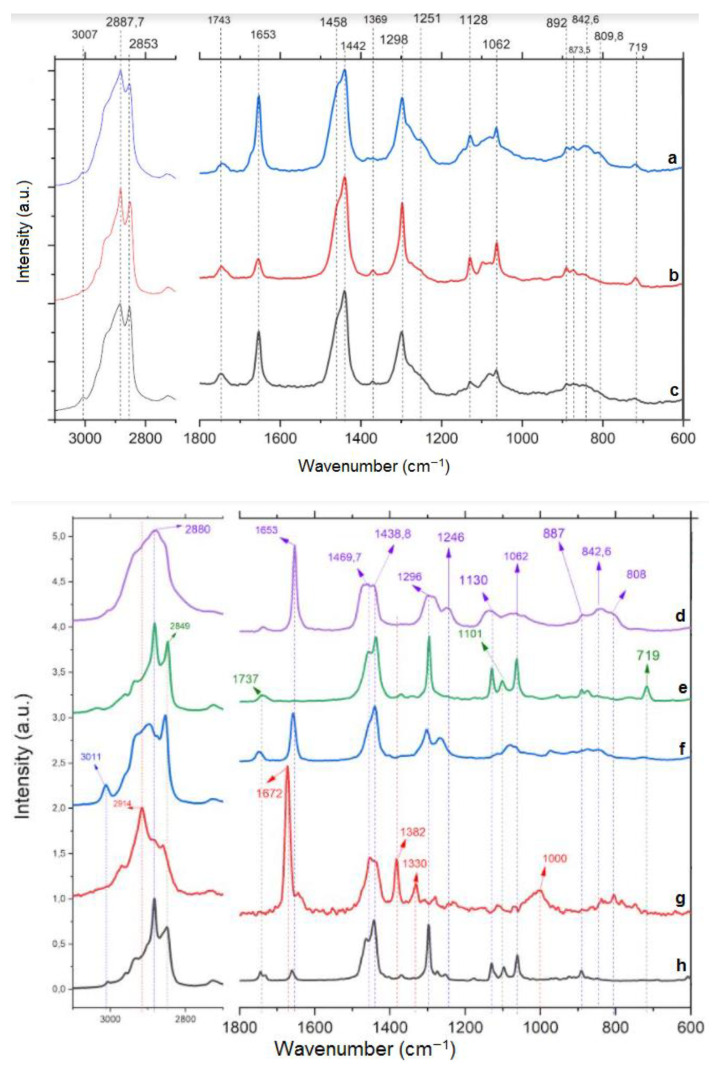
FT-Raman spectra of physical mixture of OF12 (**a**), lyophilized OF12B (**b**), lyophilized OF12 (**c**), Tween 80 (**d**), Phospholipon^®^ 80H (**e**), sesame oil (**f**), Palmarosa essential oil (**g**), and cocoa butter (**h**).

The FT-Raman spectra are shown in Figure 5. Due to the similar chemical compositions of P80H and cocoa butter, their Raman spectra are very similar. The bands at 2880 and 2849 cm^−1^ represent CH_3_ symmetric and CH_2_ asymmetric and stretching, respectively [53]. The band at 1440 cm^−1^ is related to CH_2_ bonds while the ones at 1130 and 1062 cm^−1^ refer to C-C asymmetric and symmetric stretching, respectively [54]. The band at 1101 cm^−1^ is characteristic of conformationally disordered alkyl chains with gauche defects [55]. Peaks at 1298 cm^−1^ are attributed to CH_2_ twisting and peaks between 800 and 900 cm^−1^ to CH_3_ rocking [56]. On liquid excipients, like sesame oil, bands at 800–900 cm^−1^ are related to -(CH2)n- stretching [57]. Weak peaks from 1736 to 1748 cm^−1^ are related to the carbonyl bond (C=O), which can be seen in the spectra of cocoa butter, P80H, sesame oil, and Tween 80 [58,59].

The Raman spectrum of PEO (Figure 5g) showed a broad peak at 1000 cm^−1^ associated with C-C-O stretch, commonly observed in primary monoterpenoid alcohol derivatives [60]. Peaks around 1330 are attributed to CH_2_ twisting. The olefinic unsaturation C=C stretch or the conjugated double bond system showed a relatively strong absorption peak above 1650 cm^−1^, which may be related to the presence of conjugated double bonds in geraniol, the major chemical compound present in PEO [59,61]. In addition, a peak at 2914 cm^−1^ is attributed to the symmetric C-H stretch [56]. The spectra of lyophilized NLC, blank NCL, and physical mixture presented similar profiles to each other, as well as to the lipophilic surfactant (P80H) and the solid lipid (cocoa butter) used. The strong characteristic peak of PEO (around 1650–1670 cm^−1^) can be observed in the physical mixture and lyophilized PEO-loaded NLCs. Despite the absence of PEO, this peak also appeared in the blank NLCs spectrum, with a lower intensity, because of the presence of sesame oil, cocoa butter, and Tween 80, whose spectra exhibited this peak too. Similar results were observed in the spectra of OF1 and OF4 (Supplementary Materials, Figure S3). These results may suggest the incorporation of PEO in the system and that the geraniol structure was not changed after all procedures used for obtaining the NLC. Moreover, there was no sign of interaction between the PEO and the excipients.

### 2.9. Transmission Electron Microscopy (TEM)

**Figure 6:**
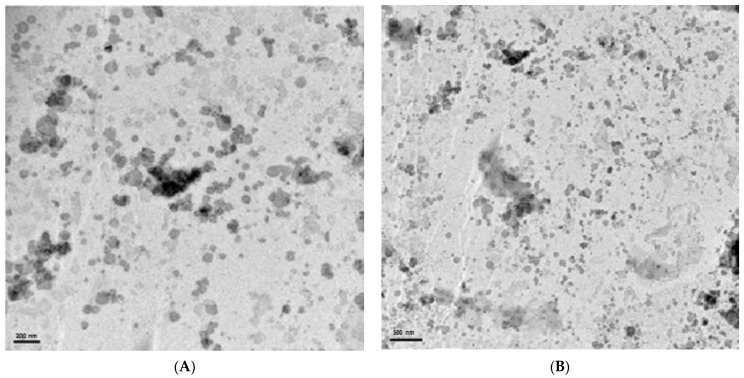
TEM micrographs of the optimized nanostructured lipid carrier containing Palmarosa essential oil OF12 at 80 kV. (**A**) 30 K magnification (scale bar = 200 nm), (**B**) 15 K magnification (scale bar = 500 nm).

The micrograph of OF12 obtained by TEM (Figure 6) confirmed the nanometric range of particles (smaller than 200 nm), which is in accordance with the DLS measurements. The nanoparticles were spherical with a narrow particle size distribution. In addition, particle agglomeration was not observed.

## 3. Materials and Methods

### 3.1. Materials

The PEO (*Cymbopogon martinii*) was purchased from the company BySamia^®^ (Perdizes, SP, Brazil). Geraniol (98% of purity) was obtained from Sigma-Aldrich^®^ (CAS: 106-24-1, Saint Louis, MO, USA). N-hexane (HPLC grade) was purchased from EM SCIENCE^®^ (CAS: 110-54-3, Gibbstown, NJ, USA) and *n*-alkanes were obtained from Sigma-Aldrich^®^ (C8-C20, Saint Louis, MO, USA). Sesame oil (*Sesamum indicum*, CAS: 8008-74-0) and Phospholipon^®^ 80H (P80H; hydrogenated soy lecithin; CAS 92128-87-5) were kindly donated by the companies Veris Brasil (Vinhedo, SP, Brazil) and LIPID Ingredients & Technologies (Ribeirão Preto, SP, Brazil), respectively. Cocoa butter was purchased from Mapric^®^ (*Theobroma cacao*, CAS: 8002-31-1, Ipiranga, Brazil) and Tween 80 from Synth^®^ (polyoxyethylene sorbitan monooleate, CAS: 9005-65-6, Diadema, SP, Brazil).

### 3.2. Chemical Analysis of Palmarosa Essential Oil

The PEO was analyzed by a gas chromatography/mass spectrometry (GC-MS) system with a quadrupole detector (Thermo Electron Corporation DSQ II, TLC, Thermo Fisher, Waltham, MA, USA) equipped with a column HP-5 (30 m × 0.25 mm), in full scan mode, for 76 min. A solution of PEO in n-hexane (1746 µg mL^−1^) was prepared and then diluted in n-hexane (5:1000) for injection (1µL) in the equipment with a split ratio of 1:10. The chromatographic conditions were as follows: the oven temperature increased from an initial temperature of 50 °C min^−1^ to 230 °C (3 °C min^−1^); the injector and detector temperatures were 250 °C and 270 °C, respectively; the carrier gas (helium) flow rate was 1 mL min^−1^; and the temperature of the ionization source was 270 °C.

The characterization was based on the retention time, which was compared with the main compounds using the Kovats retention index [62]. The PEO components were identified by comparison of the mass spectra with those of the NIST version 2.0 device library, literature, and Kovats retention index obtained from a mixture of *n*-alkanes (C_8_–C_24_). The chemical compounds were confirmed according to Adams (2007) and presented in a relative area (%) [63].

### 3.3. Development of a GC-MS Method for Quantification of Geraniol in the Nanostructured Lipid Carriers Containing Palmarosa Essential Oil

The analyses were performed using the same GC-MS system and the same conditions described before, except the temperature, which increased from 80 to 120 °C min^−1^ (3 °C min^−1^), followed by other heating from 50 °C min^−1^ to 280 °C (2 min). After lyophilization, the samples were diluted in *n*-hexane (1 mL) and 1 µL was injected in the equipment (split ratio of 1:10). The selection of ions present in the PEO and in standard geraniol was performed in TIC (total ion chromatogram) mode after 1:100 dilution in n-hexane from the initial concentration of 1746 and 1758 µg mL^−1^, respectively. In this new chromatographic condition, the running time was reduced to 20 min. From the TIC mode and using GC–MS-SIM (selected ion monitoring), the following selected ions were monitored for a more sensitive and selective quantification of geraniol in the NLC containing PEO: 41, 44, and 69 (11.50 and 14.00 min).

### 3.4. Antifungal Activity

#### 3.4.1. Determination of the Minimum Inhibitory Concentration (MIC) and Minimum Fungicidal Concentration (MFC) of Palmarosa Essential Oil

The *A. nomius* was obtained from the Collection of Reference Fungi on Health Surveillance (Fiocruz/CFRVS/) (Access number: 40010, Lot: 068740010). The fungus was cultivated in a tube containing Potato Dextrose Agar (PDA) (Neogen^®^ Co., Lansing, MI, USA) and maintained for 10 days, at 25 °C, in a BOD incubator (Ethik^®^, 411 FDP, Vargem Grande Paulista, SP, Brazil). The broth microdilution assay using a serial dilution was used to determine the MIC of PEO (M38-A, Clinical and Laboratory Standards Institute [CLSI], 2008). The wells of a 96-well microplate were filled with 5 µL of a suspension of *A. nomius* (10^5^ conidia mL^−1^), previously standardized with 0.9% saline solution in the Neubauer chamber, and 100 µL of RPMI 1640 medium (Sigma-Aldrich, Saint Louis, MO, USA) containing L-glutamine without bicarbonate and buffered with a 0.165 mol L^−1^ MOPS solution (Sigma-Aldrich, Saint Louis, MO, USA). The PEO was diluted in a sterile Tween 80 (1%, *v*/*v*) aqueous solution and concentrations from 3.91 to 4000 μg mL^−1^ were tested. The microplates were incubated in a BOD incubator, at 25 °C, for 72 h. The well with the least concentration was determined as the MIC value. The MFC was determined after the MIC test. For this, aliquots of 5.0 µL were taken from the wells corresponding to the concentration immediately above the MIC, the MIC, as well as three concentrations lower than the MIC, and added to sterile Petri dishes containing PDA medium. The plates were incubated in a BOD incubator for 24 h, at 25 °C, and the MFC was determined as the lowest concentration without visual fungal growth. All assays were carried out in triplicate.

#### 3.4.2. Inhibition of Mycelial Growth (IMG)

About 5.0 μL of the suspension containing *A. nomius* (10^5^ conidia mL^−1^) were inoculated in the center of a Petri dish containing 20 mL of PDA medium and one of the following samples: (1) 250 µg mL^−1^ (MIC previously determined) of Carbendazim (positive control, PC); (2) non-encapsulated PEO at a concentration of 250 µg mL^−1^ (MIC value); and (3) the NLC formulations (F1 to F13 and optimized formulations OF1, OF4, and OF12). The concentration of PEO-loaded NLC used in the test was higher than the PEO MIC [64], i.e., 3.0 mL of the formulations were added to their respective plate containing 17 mL of PDA medium. A Petri dish containing the medium and the microorganism without any treatment was used as the fungal control (FC). In addition, blank NLCs (negative control, NC) were also tested to check whether the excipients used in the preparation of the NLCs would not interfere in the results. All tests were performed in triplicate and incubated in a BOD incubator, at 25 °C, for 10 days. The diameter of the fungus colony was measured in two directions, at right angles to each other. The percentage of IMG was calculated using Equation (2), where *C*2 is the mean diameter of growth in the FC plate and *C*1 is the mean diameter of fungal growth in the treatment plates (PC, pure PEO, and PEO-loaded NLCs) [65]:

(2) $$\mathrm{IMG}\left(\%\right)=\frac{C2-C1}{C2}\times 100$$

### 3.5. NLC Development Based on the Design of Experiments (DoE)

The nanocarriers were prepared with food-grade solid and liquid lipids (cocoa butter and sesame oil (SO), respectively) by a melt-emulsification method [25,66]. Firstly, the aqueous phase was prepared by dispersing the aqueous surfactant Tween 80 (2.5%, *w*/*w*) in water and heating to a temperature of 75 °C. The oil phase, comprising the cocoa butter, SO, PEO, and the lipophilic surfactant P80H, was also heated to the same temperature. Then, the aqueous phase was added over the oil phase under constant agitation, at 5400 rpm, for 2 min, using an Ultra-Turrax T25 homogenizer (IKA^®^ Works, Wilmington, NC, USA). The temperature was kept at 75 °C during the process. Afterwards, the formulations were sonicated using an ultrasonic tip (model CV334 (500 W), Cole Parmer^®^, Vernon Hills, Illinois, IL, USA), set at 50% of the amplitude, for 10 min, with cycles of 59/30 s, at room temperature. Subsequently, the formulations were cooled down in the fridge for 30 min for solidification of the particles. Blank formulations (without PEO) were prepared using the same method.

After some preliminary tests, a ternary mixture design was used to evaluate the effect of some components of the formulation on the dependent variable (antifungal activity–IMG), where X_1_ is the concentration of cocoa butter, X_2_ is the concentration of PEO:SO (in a proportion of 1:3), and X_3_ is the concentration of P80H. The total amount of these components was 5.75 g for each 100 g of the formulations. The values for each component in amount (%, *w*/*w*), proportion, and pseudocomponents are shown in Table 6.

From the results obtained in the IMG test, the Scheffe model was tested in the following order: linear, quadratic, full cubic, special cubic, and special quartic order. The value of the adj R^2^ and the ANOVA results were used for validation/acceptance of the mathematical model. With a 95% confidence level (α = 0.05), the statistical significance of each term of the model was analyzed by the probability value (*p*-value) and F-value. Parameters with *p*-values less than 0.05 were considered statistically significant. In addition, the “Adeq Precision” parameter was used to assess the signal-to-noise ratio, where a value greater than 4 is desirable, indicating that the model is adequate and can be used within the planning limits. The Design-Expert software version 11 (Stat-Ease, Inc., Minneapolis, MN, USA) was used for generation and statistical analysis of the mixture design.

### 3.6. Size, Polydispersity Index, and Zeta Potential

The nanocarriers were characterized regarding the particle diameter and PDI by using the dynamic light scattering (DLS) method. The zeta potential (ζ-potential) was measured by means of electrophoretic mobility. These analyses were done in the NanoPlus/Zeta Particle Analyzer equipment (Micrometirics Instrument Corporation, Georgia, GA, USA). Before the measurements, the NLC formulations (F1 to F13 and the optimized formulations) were diluted with ultra-purified water in the proportion 1:100 [67], and it was performed at a temperature of 25 ± 1 °C and a scattering angle of 90˚. The analyses were carried out at least in triplicate.

### 3.7. Entrapment Efficiency (EE) and PEO Content (PEOC)

The method of ultrafiltration was used for determination of the EE [68]. An aliquot of 500 µL of the PEO-loaded NLC dispersions was added to Amicon ultra-filtration devices (Microcon MWCO 10kDa, Millipore Co., Billerica, MA, USA) and centrifuged at 7000 rpm for 30 min (Hettich^®^–164 Universal 320R, Tuttingen, BW, Germany). From the filtrate, 30 µL were lyophilized (Martin Christ^®^-Alpha 1-4 LD, Osterode am Harz, Germany), diluted in 1 mL of n-hexane, and then the geraniol quantification was determined by the GC-MS-SIM method (see Section 3.3). Non-filtered NLC dispersions (20 µL) were also lyophilized and diluted for total geraniol quantification. The EE was calculated by using Equation (3):

(3) $$EE\;\left(\%\right)=\left(\frac{Ctotal-Cfiltrate}{Ctheoretical}\right)\times 100$$

where *Ctotal* is the total geraniol concentration in the NLCs containing PEO (encapsulated and non-encapsulated PEO), *Cfiltrate* is the geraniol concentration found in the filtrate after ultrafiltration (non-encapsulated PEO), and *Ctheoretical* is the theoretical concentration of geraniol according to the amount of PEO present in each formulation. The PEOC was expressed as mass of PEO (in mg) per mass of formulation (in g). The analyses were carried out in triplicate.

### 3.8. In Situ Test with Brazil Nuts

The antifungal activity of PEO-loaded NLCs was also tested in Brazil nuts after inoculation with *A. nomius*. Briefly, Brazil nuts were washed with distilled water to remove the excess organic matter and sterilized in an autoclave, at 121 °C, for 20 min [69]. Fungus inoculation was performed according to Ribeiro et al. (2020), with some modifications. Each nut was inoculated in five different points with 5 μL of the suspension containing *A. nomius* (10^5^ conidia mL^−1^) and kept under a laminar flow hood for 1 h to fix the fungi [70]. After this, the contaminated nuts were treated with one of the following treatments (1.0 mL): (1) optimized NLC formulations containing PEO, (2) optimized blank NLC formulations (negative control, NC), (3) aqueous solution of Tween 80 (1%, *w*/*w*) containing 250 µg mL^−1^ of PEO, (4) aqueous solution of Tween 80 (1%, *w*/*w*) containing Carbendazim at a concentration of 250 µg mL^−1^ (positive control, PC), or 5) a 0.9% saline solution (fungal control, FC). After 2 h under the laminar flow hood for drying, the nuts were placed in sterilized tubes and incubated in a BOD incubator, at 25 °C, for 10 days. The test was performed in triplicate, where each tube contained one (treatments with non-encapsulated PEO, PC, and FC) or two (blank and PEO-loaded NLC formulations) Brazil nuts. The nuts were visually checked for deterioration.

### 3.9. Stability Study

The optimized formulations were added into glass tubes with screw caps and stored at room temperature, in the absence of light, for 90 days. Analyses of the size, PDI, ζ-potential, as well as pH, EE, IMG, and in situ antifungal test with Brazil nuts were conducted at 30-day intervals. The pH value of the NLC dispersions was determined at 25 ± 1 °C using a pH meter (Digimed Digital Model DM-22) previously calibrated with pH 4.0 and pH 7.0 standard buffers. The data were analyzed by one-way ANOVA and post-hoc Tukey’s test using the Statistica 10 (StatSoft Inc., Tulsa, OK, USA) software. The results were considered statistically different for *p*-value ≤ 0.05.

### 3.10. Fourier Transform Raman Spectroscopy (FT-Raman)

The FT-Raman analyses of optimized formulations (lyophilized PEO-loaded NLC and blank NLC), physical mixture, PEO, and raw materials (P80H, Tween 80, sesame oil, and cocoa butter) were performed using a FT-Raman spectrometer (model RAM II, Bruker, Vertex 70v, Billerica, MA, USA) at a wavelength of 1064 nm from a Nd:YAG laser, at 80 mW of power, in the wavelength range of 600–3200 cm^−1^ and resolution of 4 cm^−1^.

### 3.11. Transmission Electron Microscopy (TEM)

The morphology of the optimized formulation OF12 was observed by using a transmission electron microscope (JEM-1400, JEOL, Peabody, MA, USA). The NLC dispersion was placed onto a 400-mesh copper grid coated with carbon film (Electron Microscopy Sciences, Hatfield, PA, USA) and stained with a 5% phosphotungstic acid solution. After drying at 25 °C, for 24 h, the analysis was performed at an accelerating voltage of 80 kV.

## 4. Conclusions

The fusion-emulsification technique proved to be effective in the development of NLCs containing PEO, resulting in particles with a homogeneous size distribution and high entrapment efficiency values. The three optimized formulations proposed by the special quartic order (Scheffe model) presented small particles with a uniform size distribution, and a zeta potential suggestive of a physically stable system. Moreover, the antifungal activity test indicated that NLCs allowed the release of incorporated PEO, which could inhibit the growth of the mycotoxigenic fungus *A. nomius*. The in situ test using pre-contaminated Brazil nuts showed the potential of PEO-loaded NLC to control the deterioration caused by *A. nomius*, which was not observed for blank NLCs. In addition, even after 90 days of storage at room temperature, the NLC containing PEO maintained the physicochemical characteristics and antifungal activity. Thus, the results showed the efficiency of NLCs containing the Palmarosa essential oil, suggesting the possibility of using these systems as a biofungicide in the food industry, or even in the pharmaceutical and cosmetic industries.

## Figures and Tables

**Table 1:** Chemical composition of palmarosa (*Cymbopogon martini*) essential oil.

Peak	Compound	RIcalc	Relative Area (%)
1	NI	905	0.03
2	NI	1041	0.04
3	NI	1051	0.04
4	β-myrcene	1088	0.25
5	NI	1128	0.07
6	Trans-β-Ocimene	1134	0.30
7	Cis-β-Ocimene	1145	1.14
8	Linalool	1199	3.01
9	Geraniol	1354	80.37
10	Citral	1368	0.60
11	NI	1397	0.11
12	Geraniol ester	1477	10.85
13	Caryophyllene	1517	2.08
14	Neryl acetate	1847	0.92
15	Geranyl propionate	2042	0.18
Total amount of identified compounds			99.70
Monoterpene hydrocarbons			1.69
Oxygenated monoterpene			94.83
Sesquiterpene hydrocarbons			2.08
Others			1.10
Not identified			0.29

Note: Compounds are listed in order of elution from column HP-5; RIcalc = identification based on the retention index (RI) using a homologous series of *n*-alkane C_9_–C_24_; relative area (%): percentage of the area occupied by the compounds in the chromatogram; NI = not identified.

**Table 2:** Size, polydispersity index (PDI), zeta potential (ζ-potential), entrapment efficiency (EE), PEO content (PEOC), and inhibition of mycelial growth (IMG) of NLC formulations containing palmarosa essential oil prepared according to the mixture design.

NLC Formulations	Size (nm)	PDI	ζ-Potential (mV)	EE (%)	PEOC (mg g^−1^ of NLC)	IMG (%)
F1	236.3 ± 5.7	0.18 ± 0.01	−35.9 ± 2.7	98.7 ± 2.8	2.71 ± 0.08	43.8 ± 1.4
F2	210.4 ± 3.5	0.20 ± 0.02	−33.2 ± 0.7	89.5 ± 1.8	2.22 ± 0.04	44.7 ± 1.9
F3	269.1 ± 8.7	0.19 ± 0.03	−36.9 ± 0.2	82.8 ± 1.3	1.83 ± 0.03	29.7 ± 2.2
F4	290.8 ± 3.0	0.19 ± 0.01	−33.4 ± 0.8	78.5 ± 1.0	1.49 ± 0.02	26.9 ± 1.0
F5	231.2 ± 5.8	0.16 ± 0.02	−35.4 ± 1.0	90.7 ± 0.8	2.84 ± 0.03	98.8 ± 0.0
F6	214.9 ± 2.4	0.21 ± 0.02	−25.2 ± 0.5	86.7 ± 4.3	2.43 ± 0.12	50.6 ± 6.5
F7	173.8 ± 2.3	0.17 ± 0.02	−36.9 ± 1.3	76.1 ± 1.7	2.31 ± 0.05	60.5 ± 4.4
F8	221.4 ± 16.2	0.21 ± 0.03	−32.4 ± 0.7	86.2 ± 0.4	2.14 ± 0.01	44.2 ± 2.4
F9	193.6 ± 2.5	0.19 ± 0.01	−31.2 ± 0.5	99.9 ± 1.3	1.87 ± 0.03	24.7 ± 4.3
F10	226.5 ± 6.1	0.14 ± 0.01	−41.8 ± 1.0	95.9 ± 6.7	3.01 ± 0.21	98.8 ± 0.0
F11	198.9 ± 5.6	0.18 ± 0.02	−34.6 ± 1.4	89.5 ± 3.4	2.13 ± 0.08	45.5 ± 2.4
F12	263.2 ± 51.2	0.21 ± 0.07	−37.1 ± 1.0	96.9 ± 2.2	2.66 ± 0.06	45.1 ± 5.2
F13	191.6 ± 6.9	0.17 ± 0.02	−25.3 ± 1.1	73.6 ± 1.1	1.37 ± 0.02	31.2 ± 1.7

Note: mean ± standard deviation.

**Table 3:** Optimized formulations (OF) of NLC containing Palmarosa essential oil proposed by the Scheffe’s special quartic-order model.

Formulation	Amount (%, *w*/*w*)			Proportion (*w*/*w*)			Pseudocomponents		
	X_1_	X_2_	X_3_	X_1_	X_2_	X_3_	X_1_	X_2_	X_3_
OF1	3.897	1.247	0.606	0.678	0.217	0.105	0.330	0.552	0.118
OF4	3.833	1.258	0.659	0.667	0.219	0.115	0.259	0.564	0.177
OF12	3.858	1.254	0.638	0.671	0.218	0.111	0.287	0.560	0.153

X1 = concentration of cocoa butter; X2 = concentration of PEO:SO; X3 = concentration of P80H.

**Table 4:** Size, polydispersity index (PI), zeta potential (ζ-potential), entrapment efficiency (EE), Palmarosa essential oil content (PEOC), and inhibition of mycelial growth (IMG) of the optimized formulations (OF) containing PEO.

NLC Formulation	Size (nm)	PDI	ζ-Potential (mV)	EE (%)	PEOC (mg g^−1^ of NLC)	IMG (%)
OF1	112.0 ± 1.6	0.16 ± 0.001	−34.6 ± 2.2	101.7 ± 0.9	3.17 ± 0.03	97.3 ± 1.3
OF4	178.6 ± 2.7	0.27 ± 0.008	−32.7 ± 0.1	100.2 ± 0.8	3.15 ± 0.03	100
OF12	143.3 ± 0.8	0.18 ± 0.003	−29.1 ± 0.7	102.7 ± 2.4	3.22 ± 0.07	100

Note: mean ± standard deviation.

**Table 5:** Size, polydispersity index (PDI), zeta potential (ζ-potential), entrapment efficiency (EE), Palmarosa essential oil content (PEOC), pH, and inhibition of mycelial growth (IMG) of the optimized formulations (OFs) after 24 h, 30 days, 60 days, and 90 days of storage at room temperature.

		Time			
		24 Hours	30 Days	60 Days	90 Days
OF1	Size (nm)	112.1 ± 1.6 ^b^	125.9 ± 3.2 ^a^	118.3 ± 0.1 ^c^	123.2 ± 1.1 ^b^
	PDI	0.16 ± 0.001 ^a^	0.20 ± 0.01 ^b^	0.16 ± 0.01 ^a^	0.18 ± 0.02 ^a,b^
	ζ-potential (mV)	−34.6 ± 2.2 ^a^	−31.8 ± 1.4 ^a,b^	−30.8 ± 0.8 ^b^	−31.8 ± 0.5 ^a,b^
	EE (%)	101.7 ± 0.9 ^b^	101.6 ± 0.5 ^a,b^	100.3 ± 1.6 ^a,b^	99.2 ± 0.3 ^a^
	PEOC *	3.17 ± 0.03 ^b^	3.17 ± 0.02 ^a,b^	3.13 ± 0.05 ^a,b^	3.09 ± 0.01 ^a^
	pH	6.35 ± 0.04 ^a^	6.42 ± 0.04 ^a^	6.38 ± 0.02 ^a^	6.42 ± 0.03 ^a^
	IMG (%)	97.29 ^b^	100 ^a^	100 ^a^	100 ^a^
OF4	Size (nm)	178.6 ± 2.7 ^c^	121.2 ± 2.1 ^a,b^	119.9 ± 1.0 ^a^	125.2 ± 1.3 ^b^
	PDI	0.27 ± 0.01 ^b^	0.19 ± 0.02 ^a^	0.16 ± 0.03 ^a^	0.18 ± 0,01 ^a^
	ζ-potential (mV)	−32.7 ± 0.1 ^a^	−27.6 ± 1.1 ^c^	−29.8 ± 1.2 ^b^	−33.4 ± 0.2 ^a^
	EE (%)	100.3 ± 0.8 ^a^	100.9 ± 3.3 ^a^	100.4 ± 1.2 ^a^	100.1 ± 1.1 ^a^
	PEOC *	3.15±0.03 ^a^	3.18 ± 0.10 ^a^	3.16 ± 0.04 ^a^	3.15 ± 0.03 ^a^
	pH	6.42 ± 0.03 ^a^	6.34 ± 0.05 ^a^	6.41 ± 0.04 ^a^	6.42 ± 0.01 ^a^
	IMG (%)	100	100	100	100
OF12	Size (nm)	143.4 ± 0.8 ^b^	138.8 ± 2.3 ^a,b^	137.8 ± 1.3 ^a^	137.6 ± 2.4 ^a^
	PDI	0.18 ± 0.00 ^a^	0.17 ± 0.02 ^a^	0.17 ± 0.01 ^a^	0.18 ± 0.00 ^a^
	ζ-potential (mV)	−29.1 ± 0.7 ^a^	−27.4 ± 0.1 ^a^	−29.1 ± 1.6 ^a^	−26.9 ± 0.5 ^a^
	EE (%)	102.7 ± 2.4 ^a^	101.4 ± 3.4 ^a^	102.9 ± 1.7 ^a^	102.5 ± 2.6 ^a^
	PEOC *	3.22 ± 0.07 ^a^	3.18 ± 0.11 ^a^	3.23 ± 0.05 ^a^	3.21 ± 0.08 ^a^
	pH	6.31 ± 0.03 ^a^	6.38 ± 0.03 ^a^	6.35 ± 0.03 ^a^	6.37 ± 0.05 ^a^
	IMG (%)	100	100	100	100

Note: mean ± standard deviation. One-way ANOVA with post-hoc Tukey HSD; ^a,b,c^ are considered statistically different (*p* < 0.05). * PEOC (mg g^−1^ of NLC).

**Table 6:** Planning of ternary mixture of NLC containing Palmarosa essential oil.

Formulations	Amount (%, *w*/*w*)			Proportions (*w*/*w*)			Pseudocomponents		
	X_1_	X_2_	X_3_	X_1_	X_2_	X_3_	X_1_	X_2_	X_3_
F1	4.150	1.100	0.500	0.722	0.191	0.087	0.611	0.389	0.000
F2	4.017	0.992	0.742	0.699	0.172	0.129	0.463	0.269	0.269
F3	4.233	0.883	0.633	0.736	0.154	0.110	0.704	0.148	0.148
F4	4.450	0.767	0.533	0.774	0.133	0.093	0.944	0.019	0.037
F5	3.867	1.250	0.633	0.672	0.217	0.110	0.296	0.556	0.148
F6	3.808	1.121	0.821	0.662	0.195	0.143	0.231	0.412	0.356
F7	3.600	1.217	0.933	0.626	0.212	0.162	0.000	0.519	0.481
F8	4.017	0.992	0.742	0.699	0.172	0.129	0.463	0.269	0.269
F9	4.150	0.750	0.850	0.722	0.130	0.148	0.611	0.000	0.389
F10	3.867	1.250	0.633	0.672	0.217	0.110	0.296	0.556	0.148
F11	3.800	0.950	1.000	0.661	0.165	0.174	0.222	0.222	0.556
F12	4.150	1.100	0.500	0.722	0.191	0.087	0.611	0.389	0.000
F13	4.000	0.750	1.000	0.696	0.130	0.174	0.444	0.000	0.556

Note: X_1_ = concentration of cocoa butter; X_2_ = concentration of PEO:SO; X_3_ = concentration of P80H.

## Data Availability

The data presented in this study are available in supplementary material.

## Supplementary Materials

The following files are available online. Figure S1. Gas Chromatogram (GC-MS) of Palmarosa essential oil. Table S1. ANOVA results for the regression model. Table S2. Equation model terms in inhibition of mycelial growth (IMG). Figure S2. Images of the optimized NLC formulations (OF) after 24 h (A); 30 days (B); 60 days (C); and 90 days (D) stored at room temperature. Figure S3: FT-Raman spectra of physical mixture of OF12 (a), lyophilized OF12B (b), lyophilized OF12 (c), physical mixture of OF4 (d), lyophilized OF4B (e), lyophilized OF4 (f), physical mixture of OF1 (g), lyophilized OF1B (h), lyophilized OF1 (i), Tween 80 (j), Phospholipon^®^ 80H (k), sesame oil (l), Palmarosa essential oil (m), and cocoa butter (n).

## Sample Availability

Samples of the compounds are not available from the authors.
