# Supplementary material for: Design of Nanostructured Lipid Carriers Containing Cymbopogon martinii (Palmarosa) Essential Oil against Aspergillus nomius
Source: Molecules. 2021 Aug 10;26(16):4825. doi: 10.3390/molecules26164825 (PMC8399424; doi:10.3390/molecules26164825)
Supplement: Supplementary file 1 [file molecules-26-04825-s001.zip › molecules-1299593-supplementary.pdf]

molecules

MDPI

Supplementary Materials

Design of Nanostructured Lipid Carriers Containing *Cymbopogon martinii* (Palmarosa) Essential Oil against *Aspergillus nomius*

Denise Tiemi Uchida[1], Gabriella Ferreira Siqueira[2], Edson Marques dos Reis[3], Fábio Luis Hegeto[4], Antonio Medina Neto[4], Adriano Valim Reis[2], Marcos Luciano Bruschi[2,*], Mônica Villa Nova[2] and Miguel Machinski Júnior[1]

[1] Department of Health Basic Sciences, State University of Maringá, 87020-900, Maringá, PR, Brazil; denisetiemi13@gmail.com (D.T.U.); mmjunior@uem.br (M.M.J.)
[2] Department of Pharmacy, State University of Maringá, 87020-900, Maringá, PR, Brazil; gabisiqueiraa9@gmail.com (G.F.S.); avreis@uem.br (A.V.R.); mlbruschi@uem.br (M.L.B.); monica.villano@gmail.com (M.V.N.)
[3] Department of Chemistry, State University of Maringá, 87020-900, Maringá, Brazil; emreis@uem.br
[4] Department of Physics, State University of Maringá, 87020-900, Maringá, Brazil, hegetofl@hotmail.com (F.L.H.); ;medina@dfi.uem.br (A.M.N.)
* Correspondence: mlbruschi@uem.br; Tel.: +55 44 3011-5998

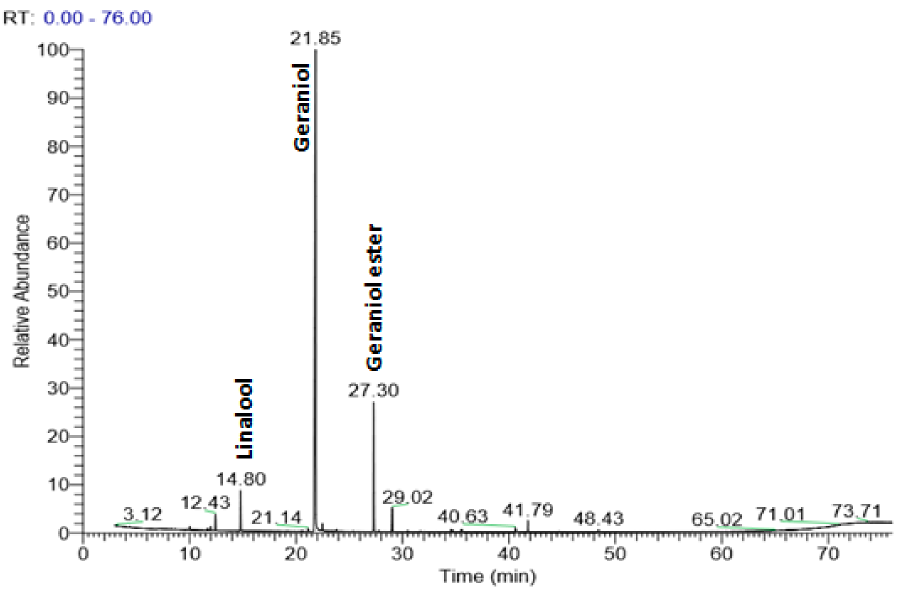

Figure S1. Gas Chromatogram (GC-MS) of Palmarosa essential oil.

Table S1. ANOVA results for the regression model.

Parameters	SS	df	MS	*F*	*p*-value
**Model**	6944.67	8	868.08	2886.87	< 0.0001
Linear mixture	5018.70	2	2509.35	8345.02	< 0.0001
X_1X_2	512.06	1	512.06	1702.90	< 0.0001
X_1X_3	25.40	1	25.40	84.45	0.0008
X_2X_3	721.76	1	721.76	2400.25	< 0.0001
$X_1^2X_2X_3$	324.31	1	324.31	1078.51	< 0.0001
$X_1X_2^2X_3$	60.17	1	60.17	200.10	0.0001
$X_1X_2X_3^2$	120.77	1	120.77	401.63	< 0.0001
**Residual**	1.20	4	0.3007		
Lack of Fit	0.1503	1	0.1503	0.4283	*0.5595
Pure error	1.05	3	0.3508		
Total	6945.87	12			

*not significant value

Note: SS – sum of square; df – degrees of freedom; MS – mean of square.

Table S2. Equation model terms in inhibition of mycelial growth (IMG).

Terms	Coefficient	Standard error	*p*-value
X_1	32.29	0.71	< 0.0001
X_2	526.29	10.96	< 0.0001
X_3	76.97	5.45	< 0.0001
X_1X_2	-757.21	18.35	< 0.0001
X_1X_3	-105.06	11.43	< 0.0001
X_2X_3	-998.96	20.39	< 0.0001
$X_1^2X_2X_3$	2477.61	75.44	< 0.0001
$X_1X_2^2X_3$	-2469.45	174.57	< 0.0001
$X_1X_2X_3^2$	3425.25	170.91	< 0.0001

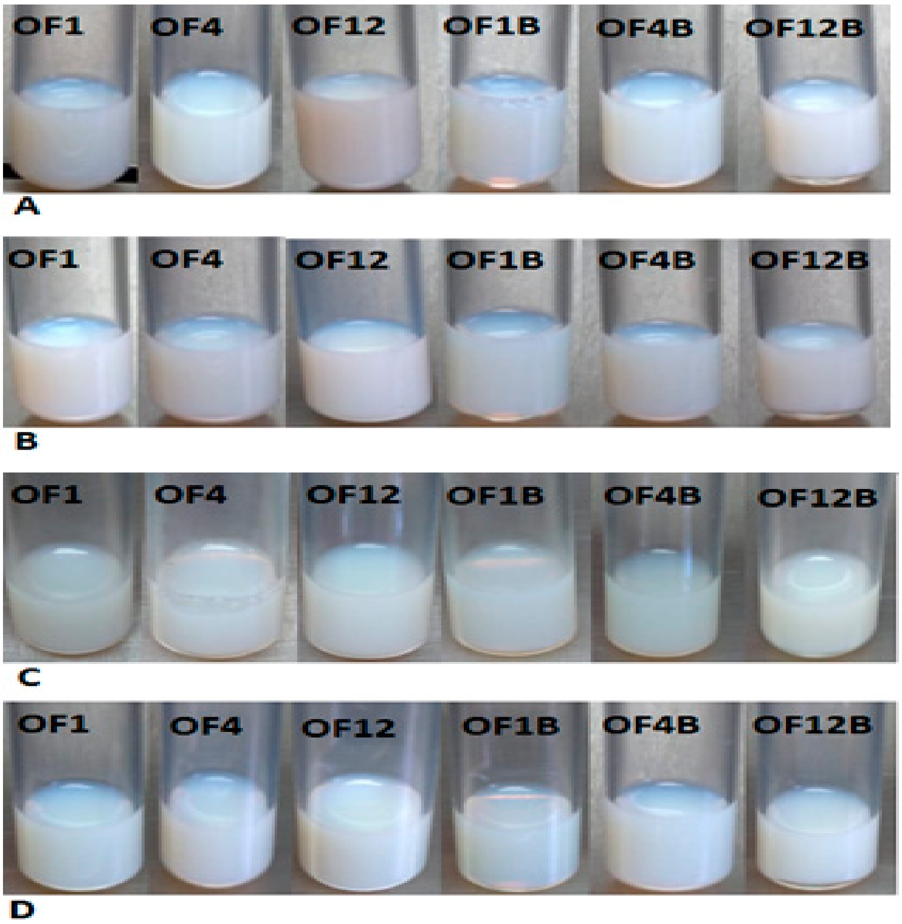

Figure S2. Images of the optimized NLC formulations (OF) after 24 h (A); 30 days (B); 60 days (C) and 90 days (D) stored at room temperature.

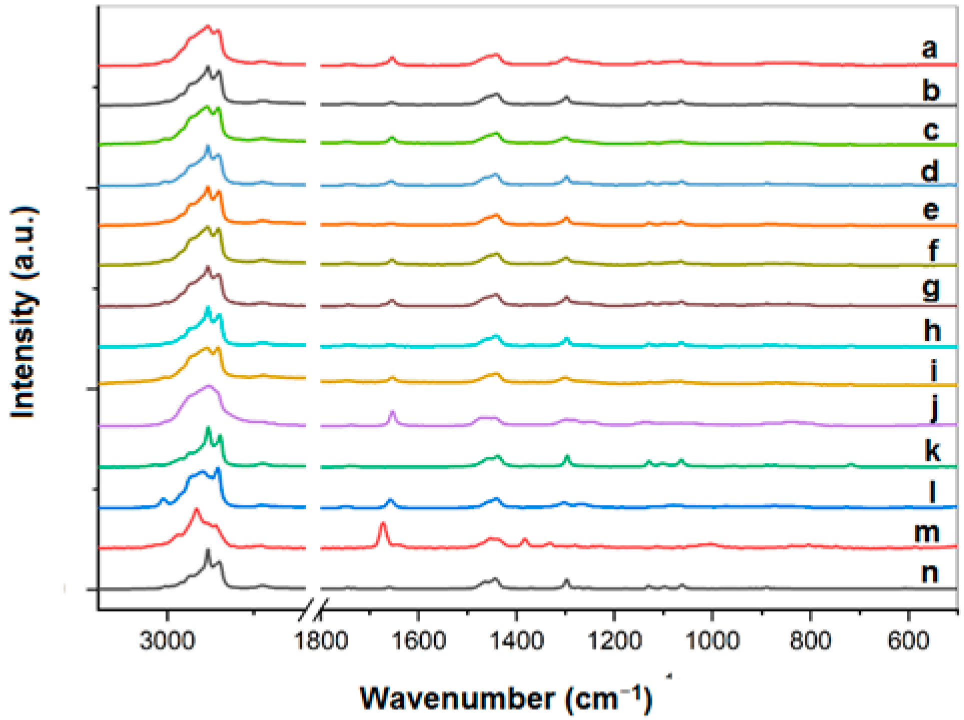

Figure S3: FT-Raman spectra of physical mixture of OF12 (a), lyophilized OF12B (b), lyophilized OF12 (c), physical mixture of OF4 (d), lyophilized OF4B (e), lyophilized OF4 (f), physical mixture of OF1 (g), lyophilized OF1B (h), lyophilized OF1 (i), Tween 80 (j), Phospholipon® 80H (k), sesame oil (l), Palmarosa essential oil (m) and cocoa butter (n).
